# Neuroradiological Insights into Visual Mental Imagery: Structural and Functional Imaging of Ventral and Dorsal Streams

**DOI:** 10.3390/brainsci16040345

**Published:** 2026-03-24

**Authors:** Saleha Redžepi, Edin Avdagić, Ajša Šahinović, Mirza Pojskić

**Affiliations:** 1 Department of Radiology, Clinical Center University of Sarajevo, 71000 Sarajevo, Bosnia and Herzegovina; saleharedzepi98@gmail.com (S.R.); ajsasahinovic@gmail.com (A.Š.); 2 Department of Neuroradiology, Clinical Center University of Sarajevo, 71000 Sarajevo, Bosnia and Herzegovina; edoavdagic@gmail.com; 3 Department of Neurosurgery, University Hospital Marburg, Philipps University Marburg, 35043 Marburg, Germany

**Keywords:** visual mental imagery, functional neuroimaging, neuroradiology, ventral visual stream, dorsal visual stream, brain connectivity, diffusion MRI, lesion studies, aphantasia, hyperphantasia

## Abstract

**Highlights:**

**What are the main findings?**

- Visual mental imagery engages ventral and dorsal stream systems in a content- and stage-dependent manner, with evidence for stream interaction that varies across paradigms and populations.
- Structural and clinico-radiological evidence is broadly consistent with disconnection frameworks, suggesting that disruption of long-range pathways (e.g., inferior longitudinal fasciculus (ILF)/inferior fronto-occipital fasciculus (IFOF)/ superior longitudinal fasciculus (SLF)) may contribute to imagery deficits beyond focal cortical damage.

**What are the implications of the main findings?**

- Stream-sensitive phenotyping (object vs. spatial imagery) and stage-aware paradigms are essential to develop interpretable neuroradiological biomarkers.
- Multimodal protocols combining structural MRI, diffusion imaging/tractography, functional connectivity, and lesion mapping can improve clinical interpretation of imagery complaints.

**Abstract:**

Visual mental imagery, the ability to generate and manipulate internal visual experiences without direct sensory input, links perception with memory, planning, and higher cognition. In this targeted narrative review, we synthesize neuroimaging and lesion evidence on the brain basis of visual imagery, with a focus on neuroradiological correlates of the ventral and dorsal visual pathways. Unlike prior cognitive neuroscience reviews that primarily emphasize functional mechanisms, this review is neuroradiology-oriented and integrates lesion patterns and white-matter disconnection to support clinico-radiological interpretation of imagery complaints. Using a dual-stream framework, we contrast ventral occipito-temporal systems that preferentially support object imagery (appearance-based features such as form, faces/objects, and color, with texture remaining under-studied) with dorsal occipito-parietal systems that preferentially support spatial imagery (relations, transformations, and navigation). Across studies, imagery recruitment is strongly task- and stage-dependent: ventral regions are most often engaged during object-focused imagery, whereas parietal regions are prominent during spatial transformation tasks, with evidence for interaction between pathways when demands require both content and spatial operations. Structural and clinico-radiological findings indicate that imagery impairment can arise from focal posterior lesions and posterior neurodegenerative syndromes but also from network disruption affecting long-range connections that support top-down access to posterior representations. Finally, emerging work on aphantasia and hyperphantasia supports a network-level view in which imagery vividness relates to how effectively higher-order systems engage visual representations. We conclude that standardized, stream-sensitive tasks and multimodal approaches combining functional and structural imaging with lesion-based evidence are key to discovering clinically actionable biomarkers of imagery dysfunction.

## 1. Introduction

### 1.1. Visual Mental Imagery as a Bridge Between Perception and Cognition

Visual mental imagery refers to the ability to generate, maintain, and manipulate quasi-perceptual visual experiences in the absence of retinal stimulation. Rather than a unitary capacity, imagery comprises partially dissociable component processes, including generation, maintenance, inspection, transformation, and evaluation, which can vary across individuals and neurological conditions. Imagery supports cognition by enabling internally generated visual content to guide memory retrieval, planning, and problem solving beyond immediate sensory input. Neuroimaging evidence suggests that visual mental imagery recruits distributed neural networks overlapping with perceptual systems, while its phenomenological variability is linked with the structure and function of the primary visual cortex. This review integrates functional and structural neuroimaging evidence (e.g., functional magnetic resonance imaging (fMRI), structural MRI, and diffusion imaging) and lesion studies with the aim to delineate the neural substrates of visual mental imagery [1].

A prominent dimension of imagery is vividness, the subjective strength, clarity, and detail of internally generated experience, which varies widely and has been linked with measurable differences in neural recruitment, particularly the degree to which imagery-related activity patterns resemble perceptual patterns within visual cortices [2,3,4]. Notably, by being intrinsically subjective, self-reported vividness is objectively unverifiable, and it limits cross-individual comparability. As such, it is susceptible to response bias (e.g., expectation) and may reflect metacognitive judgments rather than perceptual experience.

Clinically, imagery can reveal representational and control impairment that may not be captured by standard perceptual testing. Importantly, imagery provides a controlled way to probe top-down sensory recruitment because the absence of retinal input reduces feedforward drive; thus, activity in visual cortices can be more directly attributed to internally generated, feedback-driven signals. At the same time, internally generated representations remain constrained by higher-order processes (e.g., memory and attention), enabling mechanistic inference about how cognitive control shapes sensory-level activity. These properties make imagery informative for interpreting patient reports and stratifying individuals in neuroimaging and neuropsychological assessment [1,2,3].

### 1.2. Dual-Stream Model and the Neuroradiological Gap

A central organizing principle in visual neuroscience is the dual-stream model, which proposes a ventral occipito-temporal pathway biased toward object identity and features (“what”) and a dorsal occipito-parietal pathway biased toward spatial relations and visually guided action (“where/how”) [4,5,6]. However, the strictness of this distinction remains debated: newer accounts do not abandon the framework but refine it by emphasizing graded specialization, shared representations, and context-dependent interactions between streams (e.g., via attention, memory, and goal-directed control), rather than anatomical or functional independence [7,8]. This interactive view is particularly relevant for visual mental imagery, where internally generated content is expected to recruit ventral systems more strongly when appearance-based object features are prioritized, and dorsal systems when spatial relations or transformations are required, with variable early visual cortex involvement depending on task demands and individual differences [1,9,10,11].

Given the methodological diversity of the imagery literature, our review is a targeted narrative synthesis rather than a formal systematic review. From a neuroradiology perspective, a unified stream- and network-based scaffold is needed to translate heterogeneous readouts (e.g., activation, connectivity, atrophy, and tract integrity) into coherent clinico-anatomical interpretations. Without such a framework, “reduced” ventral/dorsal engagement or posterior abnormalities can remain clinically ambiguous (local dysfunction vs. disconnection vs. compensation), limiting reporting consistency and diagnostic inference. Studies were selected using predefined inclusion principles focused on neuroradiological interpretability (i.e., paradigms that dissociate object vs. spatial imagery and/or imagery stages, and evidence that links functional findings with structural, connectivity, or lesion anatomy), prioritizing convergent patterns reported across independent tasks and cohorts. Accordingly, we organize heterogeneous results around a dual-stream, stage-aware framework to extract clinically actionable insights, namely, which posterior cortical territories and long-range pathways are most plausibly implicated when patients report selective object vs. spatial imagery disturbance and when symptoms suggest network-level disconnection beyond focal cortical damage [1,2,3,12,13]. For neuroradiologists, a unified stream- and network-based scaffold is needed to translate heterogeneous imaging readouts (cortical involvement, tract integrity, and connectivity patterns) into consistent clinico-radiological interpretations of imagery complaints, particularly when symptoms appear disproportionate to the visible lesion core.

### 1.3. Clinical Relevance

Imagery complaints can index posterior network dysfunction that may be missed by brief perceptual screening, making them relevant to neurology, neuropsychology, and neuroradiology.

#### 1.3.1. Neurology (Lesions, Neurodegeneration, and Tumors)

Posterior lesions (e.g., stroke and tumor-related mass effect/edema) can produce stream-biased imagery deficits depending on ventral occipito-temporal vs. dorsal occipito-parietal involvement and network disruption. Posterior cortical atrophy (PCA) provides a natural model of stream-weighted vulnerability, with imaging patterns that often differentiate occipito-parietal from occipito-temporal involvement [14].

#### 1.3.2. Neuropsychology (Perception-Imagery Dissociations)

Perception and imagery can dissociate, and imagery can fractionate into object-based vs. spatial components, aligning with ventral vs. dorsal weighting [3]. Such dissociations support a network-based interpretation when patient complaints are disproportionate to basic perceptual findings.

#### 1.3.3. Neuroradiology (Network Lesions, Tracts, and Biomarkers)

A disconnection perspective is particularly relevant: long-range pathways that support ventral and dorsal processing (e.g., ILF and SLF) can contribute to imagery deficits beyond focal cortical damage [12,13]. Individual differences at the extremes of vividness (aphantasia/hyperphantasia) further motivate the search for imaging markers of network organization and top-down access to posterior visual representations [15].

Accordingly, we summarize candidate neuroradiological biomarkers of imagery dysfunction across gray matter, white-matter disconnection, and network connectivity (Section 7.2).

The aim of this narrative review is to synthesize current functional and structural neuroimaging evidence on visual mental imagery, with a specific focus on neuroradiological correlates of the ventral and dorsal visual streams, integrating findings from fMRI, structural MRI, diffusion imaging and lesion studies.

To guide this synthesis, we address four questions:

1. Stream specificity: Which functional and structural imaging findings most consistently differentiate object imagery (ventral-biased) from spatial imagery (dorsal-biased) across tasks and populations?
2. Communication and interdependence: What evidence from task-based and resting-state connectivity, as well as diffusion tractography, supports interaction between ventral and dorsal systems during imagery, and what anatomical pathways plausibly mediate this interaction?
3. Perception–imagery parallels and dissociations: Under what conditions do perceptual and imagery deficits co-occur or dissociate in lesion and disease states, and how can neuroradiology adjudicate competing explanations (local damage vs. disconnection vs. compensatory reorganization)?
4. Individual differences and clinical translation: What does emerging neuroimaging evidence on aphantasia and hyperphantasia imply for dual-stream models, and how might these insights inform neuroradiological assessment of patients reporting imagery disturbances?

## 2. Conceptual Framework: Visual Mental Imagery and Dual Visual Streams

Visual mental imagery refers to the ability to generate and manipulate quasi-perceptual experiences in the absence of corresponding external sensory input. Contemporary neurocognitive models increasingly treat imagery as an active constructive process that recruits (to varying degrees) parts of the visual system and its control networks, rather than as a unitary “picture-like” phenomenon. A dual-stream perspective is particularly useful for structuring this literature because it offers an anatomically grounded way to distinguish what is being imagined (object features, identity, and appearance) from where/how imagined content is positioned, transformed, and used for spatial reasoning. Critically, however, imagery does not map onto ventral and dorsal pathways in a one-to-one fashion: the same task can draw on multiple representational codes, and the relative weighting of ventral and dorsal contributions depends on stimulus class, task demands, strategy, and individual differences in imagery vividness and control.

### 2.1. Terminology: Object vs. Spatial Imagery

In the imagery literature, the distinction between object imagery and spatial imagery is often used to describe two partially dissociable representational emphases. This division is not merely semantic; it is motivated by converging evidence from cognitive neuropsychology, behavioral dissociations, and neuroimaging, suggesting that imagery can privilege either the appearance and intrinsic features of objects or the spatial relationships, transformations, and coordinate-like properties that support action and navigation.

In experimental terms, object imagery is typically probed using tasks that require participants to generate or compare object-specific features (e.g., feature verification, property judgments, category-specific imagery, or vividness ratings tied to surface attributes). Although object imagery can include spatial aspects (objects exist somewhere), its hallmark is the prioritization of surface and form properties that are most naturally linked with the ventral visual pathway.

Spatial imagery, by contrast, emphasizes the geometry of relations and transformations: imagining where objects are, how they are oriented, how they would look after a rotation, how to traverse an environment, or how multiple elements are arranged relative to one another. Spatial imagery is commonly studied through mental rotation paradigms, imagined perspective changes, spatial updating, route planning, or relational judgments. Classic behavioral work on mental rotation demonstrates that reaction time (RT) scales with angular disparity, supporting the idea that spatial imagery often entails analog-like transformations rather than purely propositional reasoning [16]. Imaging evidence further indicates that spatial imagery tasks frequently recruit dorsal parietal circuitry, with additional involvement of frontal control systems depending on complexity and strategy [17].

Importantly, this object–spatial distinction should be treated as a dominant representational mode rather than a strict dichotomy. Consequently, a dual-stream framework is best conceptualized as a structured hypothesis about relative weighting: object imagery should preferentially engage ventral regions and ventral connectivity when surface/form detail is central [18,19].

**Figure 1:**
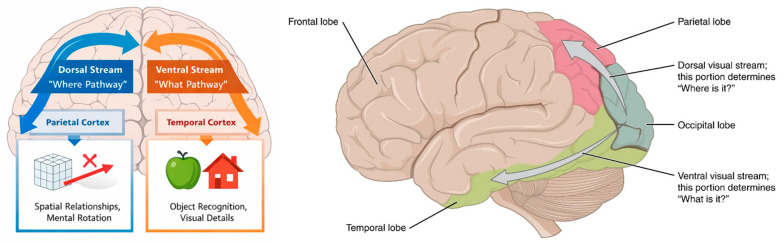
Dual-stream model and imagery domains: The dual-stream model of visual processing, highlighting the dorsal “where” pathway and the ventral “what” pathway along with the associated imagery domains. Adapted from OpenStax, *Anatomy and Physiology*, licensed under CC BY 4.0; attribution to OpenStax (Rice University). Available online: https://oerpub.github.io/epubjs-demo-book/content/m46557.xhtml (accessed on 20 February 2026). Symbols/icons indicate example task content: the cube and arrow (with X) denote spatial transformation, whereas the apple/house icons denote object-based imagery.

Figure 1 summarizes the dual-stream framework and the proposed mapping of object vs. spatial imagery components onto ventral and dorsal visual pathways, highlighting task-dependent interaction across streams (Figure 1).

### 2.2. Ventral Stream Contributions to Object Imagery

The ventral visual stream, extending from the occipital cortex to the ventral temporal regions, is classically linked with object identification and detailed feature processing. In imagery, ventral stream involvement is most clearly supported when tasks explicitly require the reinstatement of category and feature-specific representations (e.g., faces, places, and objects; color; and fine-grained form) [20,21].

Form and category representations. From a neuroradiological standpoint, the ventral temporal cortex is particularly relevant because it contains high-level feature maps and category-selective regions that can be re-engaged during the internal generation of visual content. The interpretive value here is twofold: ventral regions support appearance-based representations and are therefore expected to be vulnerable in object imagery deficits [3].

#### 2.2.1. Color Imagery

Color is a particularly informative test case because it is a surface attribute with well-characterized cortical correlates (including visual area V4/human V4 (V4/hV4)). Evidence indicates that internally generated color experiences can converge with perceptual color representations in the ventral visual cortex and that patterns in hV4 can predict behavioral performance in color imagery tasks, positioning ventral stream regions as plausible perceptual “hubs” for both externally driven and internally generated color content [22].

#### 2.2.2. Texture Imagery as an Under-Studied Dimension

In contrast to color and broad category effects, texture remains comparatively under-represented in dedicated imagery paradigms, despite its central role in real-world object perception (e.g., material identification, surface quality, and affordance inference). Neuroimaging and neuropsychological work on perception has shown separable processing of texture/surface properties vs. geometric form within the ventral occipito-temporal cortex, including evidence that surface and form can dissociate across regions and across patients with visual agnosia profiles [23]. More recent work further emphasizes that “texture-like” feature representations are distributed across the visual cortex and can serve as meaningful representational axes for object processing [24].

### 2.3. Dorsal Stream Contributions to Spatial Imagery

Among spatial imagery paradigms, mental rotation provides the clearest and most replicated functional imaging signature: increased activation in the parietal cortex (often centered around intraparietal sulcus and adjacent superior/inferior parietal regions), with modulation according to task demands and rotation parameters [17,19].

Mental rotation and spatial transformations. Neuroimaging syntheses show that mental rotation robustly engages intraparietal and adjacent parietal regions, with additional recruitment of premotor and prefrontal areas depending on strategy and stimulus type [17]. Empirically, task-based fMRI studies confirm a “parietal core” for mental rotation across different stimulus classes, consistent with a dorsal stream computational role in implementing the spatial transformation itself [19]. Notably, evidence also supports interactions between dorsal and ventral pathways during mental rotation, especially when stimulus properties increase representational demands, implying that spatial imagery tasks can recruit ventral representations in parallel with dorsal transformation mechanisms [18].

Spatial relations, navigation, and scene construction. Beyond rotation, spatial imagery extends to representing environmental layout and navigational affordances. Scene- and place-related processing has been linked with specialized regions in the ventral/medial temporal cortex (e.g., parahippocampal place area—PPA), as well as broader networks supporting spatial context [25]. Work on imagination and scene construction further implicates coordinated hippocampal–neocortical interactions, suggesting that coherent spatial frameworks for imagery depend on relational memory systems in addition to dorsal parietal computations [26,27]. Complementing this, evidence has proposed a role for the parahippocampal cortex in representing spatial structure and “space-defining” properties, highlighting that spatial representation is not exclusively dorsal in anatomical terms [28].

### 2.4. Implications for Neuroradiological Interpretation

A central conceptual question for neuroradiological synthesis is the extent to which imagery “reuses” perceptual machinery. The most influential evidence for perception–imagery parallels comes from findings that imagery can recruit the visual cortex, sometimes including early visual areas, and can do so with retinotopically meaningful organization [20,21].

However, parallels are not identity, and several neuroradiologically relevant considerations motivate caution:

1. Overlap is task-dependent and increases when fine-grained detail is required [21];
2. Imagery places a greater burden on top-down control, especially in spatial transformation tasks [17,18];
3. Neuropsychological dissociations show that imagery and perception can fractionate, including separable “visual/object” vs. “spatial” imagery impairment [3].

Taken together, these points motivate a network-based view in which ventral and dorsal pathways provide a principled scaffold for predicting vulnerability to lesions and disconnections, while the degree of perception–imagery coupling depends on task demands and available control mechanisms.

## 3. Functional Neuroimaging of Visual Mental Imagery

Functional neuroimaging, most prominently fMRI, shows that visual mental imagery recruits distributed systems that partially overlap with perception while relying more on top-down control and memory-based construction [1,2,29,30]. Building on dual-stream accounts [4,5,6,7,8], current work targets which representations are reinstated (object vs. spatial), which stages are engaged (e.g., generation, maintenance, and transformation), and how networks coordinate internally generated content. Because imagery depends on feedback and long-range integration, it is especially sensitive to network disruption from lesions, disconnection, and neurodegeneration (Section 4 and Section 5). This section provides a qualitative synthesis that emphasizes convergence, defined as recurring activation or connectivity patterns observed across independent studies despite differences in paradigms and analytic approaches.

### 3.1. fMRI Paradigms and Design Choices

#### 3.1.1. Imagery vs. Perception Contrasts

A key design choice is whether imagery is contrasted with perception, rest/baseline, or an active control task. Imagery–perception contrasts test shared machinery: imagining can recruit feature- and category-sensitive regions also engaged during seeing, typically with lower amplitude and greater reliance on top-down systems [1,2,9,20,21,29,30]. Ganis et al. reported substantial posterior overlap alongside stronger fronto-parietal engagement during imagery under matched demands [31]. Differences in temporal dynamics (e.g., variable imagery onset/duration) can complicate direct imagery–perception fMRI comparisons. Interpretation also requires caution: reduced imagery blood-oxygen-level dependency does not imply absent representation and may reflect lower sensory gain, temporal jitter, or reliance on higher-level codes rather than early visual detail [2,10,21,29,30]. These issues motivate objective task constraints, trial-wise vividness ratings, and connectivity/representational analyses (Section 3.4).

#### 3.1.2. Block vs. Event-Related Designs

Block designs increase power for weaker imagery effects but can conflate generation, maintenance, inspection, and decision making. Event-related designs can separate stages (cue → delay → probe) and enable the trial-wise modeling of imagery success. Hybrid designs are common. For neuroradiological studies of imagery deficits, event-related or hybrid designs that isolate stages are generally preferable; block designs are useful when maximizing detection is the priority.

For neuroradiological studies of imagery deficits, event-related or hybrid designs that separate generation/maintenance/transformation and enable trial-wise modeling are generally preferable, with block designs being reserved for maximizing detection power when effects are weak.

#### 3.1.3. Imagery Generation Posterior Overlap Alongside Maintenance vs. Transformation

A neuroradiologically useful conceptual refinement is to distinguish imagery stages:

1. Generation: Initiating an internal image from memory/instruction.
2. Maintenance/inspection: Stabilizing the image and extracting features.
3. Transformation: Manipulating the image (e.g., rotation, scaling, reconfiguration, and navigation).

These stages are commonly isolated with cue–delay–probe event-related paradigms (generation at cue, maintenance during delay, and inspection at probe) [20,21,31], whereas transformation is typically probed with mental rotation/manipulation tasks [16,17,18,19].

Stage separation matters because different stages weight ventral and dorsal systems differently: object-feature inspection tends to emphasize ventral representations, spatial manipulation emphasizes dorsal parietal systems, and generation tends to amplify control and memory demands [1,3,17,18,19]. Importantly, a person can show “intact” ventral representations during maintenance but impaired generation if top-down access routes are compromised—an interpretive pattern that becomes central when integrating functional imaging with diffusion and lesion evidence later in the review.

#### 3.1.4. Measures: Vividness Ratings and Objective Performance

Imagery is quantified via vividness ratings and/or objective performance (accuracy/RT). Vividness of Visual Imagery Questionnaire (VVIQ) is widely used, and many studies collect trial-wise vividness to capture within-subject variability [32]. Vividness relates to measurable neural markers, including early visual cortex engagement [33], and can be modulated by attention and competitive suppression effects (including negative blood-oxygen-level-dependent (BOLD) patterns) [34]. Objective measures (e.g., feature verification, rotation costs, and spatial precision) strengthen inference but should minimize verbal/semantic shortcuts, especially for surface-property tasks (Section 3.2.3).

**Figure 2:**
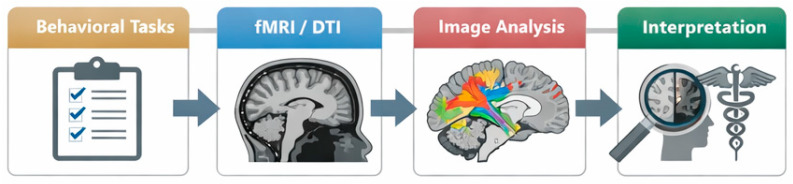
Multimodal methods-to-meaning pipeline, from task-based data collection through analysis to final interpretation. Created by the authors for this review. Colors in the tract illustration are schematic and used only to distinguish major association pathways (e.g., ventral occipito-temporal and dorsal parieto-frontal routes); they do not represent a tract-specific atlas or a standardized color code.

To facilitate neuroradiology-oriented interpretation across heterogeneous paradigms, Table 1 summarizes what each imaging modality measures and its inference strengths/limitations for ventral/dorsal questions (Table 1). Figure 2 complements Table 1 by illustrating how methodological choices and imaging readouts translate into neuroradiological inference about ventral/dorsal stream involvement and stream interaction (Figure 2).

### 3.2. Ventral Stream Activation During Object Imagery

Object imagery engages ventral occipito-temporal systems most clearly when tasks require appearance-based representations rather than semantic knowledge [1,2,9,29,30]. Across paradigms, frequently implicated loci include the fusiform/inferior temporal (IT) cortex, the lateral occipital complex (LOC), and feature-sensitive regions supporting surface attributes such as color.

#### 3.2.1. Fusiform and Inferior Temporal Regions

Imagining faces and places activates corresponding category-selective regions [9]. Ishai et al. further showed that imagery of famous faces engages a distributed network including the ventral temporal cortex, modulated by attention and memory demands, consistent with top-down reactivation during object imagery [35].

Ventral activity during imagery may index representational reinstatement when tasks require appearance-specific detail but may also partly reflect top-down semantic or mnemonic associations when imagery demands are weak or strategies are more verbal/semantic [1,2,35]. Clinically, this supports the prediction that ventral lesions or access-network disruption can selectively degrade object imagery vividness/accuracy [3,14].

#### 3.2.2. Lateral Occipital Complex (LOC)

The LOC, a key node for object perception and shape processing [36], is commonly recruited when tasks require inspection or comparison of object form. This supports the view that mid-level structural representations can be reinstated by top-down signals, with recruitment varying by the granularity of object information required.

#### 3.2.3. Feature-Specific Object Imagery: Color, Shape, and Texture

Color imagery provides a relatively specific assay of ventral feature reinstatement. hV4 activity patterns can predict behavioral performance in object color imagery, supporting the interpretation that imagery can reinstate perceptual feature codes beyond semantic labeling [22]. Shape and form imagery often engages ventral occipito-temporal regions (including the LOC and the inferior temporal cortex—IT), while additional fronto-parietal recruitment typically reflects control demands (e.g., generation and maintenance) rather than representational content per se [31]. Texture imagery remains comparatively under-studied, likely reflecting both a genuine gap and a methodological challenge: texture is difficult to isolate from shape and semantic labeling and therefore requires tightly constrained paradigms and objective checks [23,24]. Perceptual evidence supports separable texture/form processing [23] and distributed “texture-like” representational dimensions across the visual cortex [24], motivating imagery tasks that target material/surface properties while minimizing non-depictive strategies. In clinical neuroradiology, new complaints of impaired object/face imagery should prompt targeted scrutiny of the ventral occipito-temporal cortex (fusiform/IT/LOC vicinity) and their connecting pathways; when conventional MRI shows limited cortical destruction, consider describing the pattern as consistent with disconnection (see Section 5.1).

### 3.3. Dorsal Stream Activation During Spatial Imagery

Spatial imagery tasks that require transformations and relational computations reliably recruit dorsal occipito-parietal systems [4,5,6,7], most consistently involving intraparietal sulcus/superior parietal lobule (IPS/SPL) and often extending to precuneus and premotor regions depending on strategy and demand.

#### 3.3.1. Mental Rotation and Transformation Demands

Mental rotation shows the classic RT–angular disparity relationship [16]. Meta-analytic evidence indicates robust parietal engagement during rotation, often scaling with transformation demands [17], and empirical fMRI confirms a parietal “core” mechanism across stimulus classes [19]. Mental rotation frequently elicits bilateral parietal recruitment, which limits strict hemispheric inference, reinforcing a dorsal “core” while cautioning against overly strict single-hemisphere or single-node interpretations [17,19]. Rotation can also recruit ventral systems when object identity/complex form must be preserved, consistent with dorsal–ventral interaction [18].

#### 3.3.2. Spatial Context, Navigation, and Precuneus Involvement

Spatial imagery often extends beyond rotation to the construction and maintenance of broader spatial frameworks, imagining a scene layout, planning a route, or situating objects within contextual space. Place/scene processing has identifiable cortical anchors [25], and imagination-based paradigms implicate hippocampal–neocortical interactions in constructing coherent spatial contexts [26,27]. Complementary evidence suggests a role for the parahippocampal cortex in representing spatial structure and “space-defining” properties [28], indicating that spatial imagery is distributed across dorsal parietal systems and ventral/medial temporal circuits. Within a dual-stream framing, dorsal regions are especially implicated in transformations and spatial attention/selection, while medial temporal and ventral systems contribute stable contextual frameworks that spatial operations act upon. Medial temporal regions (hippocampus/parahippocampal cortex) provide relational scene/context representations that interact with ventral content codes and dorsal transformation/attentional mechanisms; thus, they complement rather than replace the dorsal–ventral streams [26,27,28].

Clinically, prominent difficulty with spatial visualization (e.g., mental rotation and layout/navigation imagery) should direct reporting toward dorsal occipito-parietal territories (IPS/SPL/precuneus) and parieto-frontal connections; bilateral recruitment in functional studies further supports cautious, network-level interpretation rather than a single-node account (see Section 5.1).

### 3.4. Functional Connectivity and Stream Interaction

Because imagery is initiated and constrained by top-down signals, connectivity analyses are central to its mechanism. Task-based work supports coordinated dorsal–ventral processing during demanding imagery (e.g., mental rotation requiring both transformation and object constraints) [18]. Resting-state and task-based studies further suggest that vividness relates to network organization and coupling, particularly the efficiency with which higher-order systems engage posterior visual representations [37,38,39]. Findings across aphantasia/hyperphantasia cohorts are broadly consistent with this network-level account, highlighting variability in coupling patterns across paradigms and populations [37,38,39].

### 3.5. Neuroradiological Take-Home (Functional Imaging)

In practice, task-fMRI findings are most informative when interpreted as content- and stage-dependent network recruitment rather than as a single imagery “center”. Apparent absence of ventral (object) or dorsal (spatial) engagement should be interpreted together with structural imaging and tract integrity to distinguish local dysfunction from disconnection or compensatory reorganization. When available, combining task activation with resting-state coupling can help contextualize whether reduced imagery reflects weakened network access vs. reduced representational fidelity.

## 4. Structural Neuroimaging and Connectivity

Structural neuroimaging complements fMRI by localizing posterior gray-matter nodes and white-matter pathways implicated in imagery. In this review, structural and diffusion findings are treated as independent mechanistic insights (node/tract necessity and disconnection mechanisms) that also constrain/interpret functional results, particularly when symptoms are disproportionate to focal cortical damage. Accordingly, structural and diffusion evidence is used here to specify which nodes and pathways are plausibly necessary for imagery and to explain cases where symptoms exceed what task-fMRI activation alone would predict.

Clinically actionable neuroradiology. When imagery disturbance is suspected, structural interpretation should prioritize:

1. Posterior cortical involvement within ventral occipito-temporal (object imagery) vs. dorsal occipito-parietal regions (spatial imagery);
2. Assessment of long-range association pathways supporting access and integration (ILF/IFOF for ventral-biased phenotypes; SLF for dorsal control/manipulation);
3. A network/disconnection interpretation when symptoms exceed the visible cortical lesion extent.

### 4.1. Structural MRI: Focal Lesions and Grey-Matter Correlates

#### 4.1.1. Lesion Mapping Logic and Clinico-Anatomical Inference

Voxel-based lesion–symptom mapping (VLSM) provides voxelwise links between tissue damage and imagery outcomes, improving anatomical specificity beyond descriptive case reports [40]. Morphometry (VBM/cortical thickness) can additionally relate posterior atrophy patterns to imagery measures, particularly in posterior neurodegenerative syndromes.

#### 4.1.2. Ventral Damage and Object Imagery Deficits

The most consistent structural prediction from a dual-stream framework is that damage to ventral occipito-temporal regions should disproportionately affect object imagery, especially when imagery tasks demand visual appearance properties (form, face/object identity, and surface attributes). Early neuropsychological case work supports this logic. For example, classic reports describe loss of mental imagery following occipital/occipito-temporal vascular lesions, suggesting that damage to posterior visual cortices can impair the ability to generate or inspect depictive internal representations [41].

More specifically, a detailed case study of a mental imagery deficit following posterior cerebral artery infarction and possible anoxic insult provided evidence that imagery can be selectively degraded, consistent with vulnerability of posterior visual representational systems and/or access routes [42]. At present, this ventral lesion-object imagery link is supported mainly by well-characterized single-case and small-series reports, while larger systematic lesion–symptom mapping focused specifically on object imagery remains comparatively limited.

From a neuroradiological perspective, interpretation should consider feature/task dependence (loss of depictive detail with preserved semantic knowledge) and disconnection (impaired access to ventral representations despite partial cortical preservation).

#### 4.1.3. Parietal Damage and Spatial Imagery Deficits

A complementary prediction is that damage to dorsal occipito-parietal regions should disproportionately affect spatial imagery, particularly tasks requiring transformations, coordinate computations, and spatial working memory operations. Although the spatial imagery literature includes heterogeneous paradigms (e.g., mental rotation, imagined navigation, and spatial updating), a recurring pattern is that parietal lesions can impair imagery-based spatial computations even when object knowledge and verbal reasoning remain relatively preserved (conceptually consistent with the dorsal stream’s role in transformation and relation processing; Section 2 and Section 3). Clinical neuropsychology has long leveraged these dissociations; neuroradiologically, the practical implication is that parietal infarcts, tumors, or degenerative involvement of posterior parietal cortices should raise suspicion for deficits in spatial imagery tasks (e.g., mental rotation, layout reconstruction, or imagined coordinate judgments) even if basic object recognition is less affected.

#### 4.1.4. Neurodegeneration as a Natural Model: Posterior Cortical Syndromes (PCA)

Posterior cortical atrophy provides a natural model of progressive posterior network involvement, often with dorsal-leaning visuospatial vs. ventral-leaning object/word/face profiles early in the course [43,44,45]. For neuroradiology, stream-biased atrophy patterns can contextualize imagery complaints and support syndrome-level interpretation. Illustrative vascular cases (e.g., bilateral PCA stroke with profound imagery loss) emphasize posterior hub vulnerability [46].

### 4.2. Diffusion Imaging and Tractography: The Disconnection Perspective

#### 4.2.1. Why White Matter Matters for Imagery

A disconnection framework holds that cognitive symptoms can arise from disruption of white-matter pathways, not only from focal cortical damage. This is neuroradiologically relevant because deficits may exceed what is expected from the lesion core and may occur despite apparently preserved “key” cortex on conventional MRI [47,48]. Imagery is particularly sensitive to disconnection because it depends on effective top-down access from control and memory systems to posterior representational cortices; when these routes are disrupted, imagery can fail despite partial cortical preservation. Notably, tract-level implications (e.g., ILF/IFOF/SLF) are discussed here mainly as mechanistic inferences from diffusion/tractography and disconnection frameworks, as systematic tract-specific lesion–symptom evidence for imagery outcomes remains limited. Interpretation of tract-based findings should be cautious, as diffusion MRI is model-dependent and can be affected by crossing fibers and reconstruction uncertainty, which may lead to false-positive/false-negative tract inferences. For neuroradiological reporting, tract findings are best phrased as “tract involvement consistent with disconnection” rather than as tract-specific causality (see Section 5.1).

#### 4.2.2. Ventral Pathways: ILF and IFOF (and Temporal Connections)

Within a dual-stream account, ventral imagery functions, especially object-feature imagery, should depend on white-matter systems connecting occipital visual cortices with temporal representational regions and frontal control systems.

1. Inferior longitudinal fasciculus (ILF)

The ILF supports occipito-temporal connectivity relevant to ventral representations; reduced integrity may present as disproportionate object imagery difficulty even with limited macroscopic cortical damage [12].

2. Inferior fronto-occipital fasciculus (IFOF)

The IFOF provides long-range occipito-frontal integration; disruption may weaken top-down access to posterior representations and broaden imagery complaints [49,50].

More recent synthesis work further discusses the IFOF’s complex anatomy and functional implications, underscoring that its organization remains an active topic and that it likely supports high-level integration between posterior sensory representations and frontal systems [50].

From a neuroradiological standpoint, the key implication is that ventral stream imagery symptoms may reflect impaired fronto-occipito-temporal communication rather than purely local ventral cortical damage. In practice, diffusion MRI abnormalities affecting the ILF/IFOF may help explain why some patients report disproportionate difficulty in “picturing” objects or faces even when the ventral cortex does not show extensive macroscopic injury.

#### 4.2.3. Dorsal Pathways: SLF and Parieto-Frontal Connections

Spatial imagery, especially transformation-heavy operations, is expected to rely strongly on dorsal parieto-frontal circuitry. The SLF supports parieto-frontal control relevant to spatial manipulation; disruption may manifest as impaired transformation efficiency in spatial imagery tasks [13].

In dorsal imagery tasks such as mental rotation and spatial updating, SLF integrity provides a plausible structural substrate for sustaining spatial working memory and implementing top-down selection over spatial representations.

A broader tract-level view also suggests that dorsal imagery deficits may arise from disruption of parietal connections that support integration across spatial attention, transformation, and motor planning systems. This reinforces the neuroradiological principle that “parietal lesion—spatial imagery deficit” is often an oversimplification; in many cases, the decisive factor may be which dorsal connections are interrupted and how strongly they compromise parieto-frontal coordination.

#### 4.2.4. Disconnection Explains Symptoms Better than a Single Cortical Point

Imagery deficits can reflect network disruption, damage to posterior nodes and/or interruption of the white-matter pathways that enable top-down access and inter-stream coordination, rather than a single “critical” cortical locus. Clinically, this helps explain disproportionate symptoms in stroke, tract-related deficits in tumor/neurosurgical contexts, and the progressive widening of deficits in neurodegeneration [51,52]. Practically, when imagery complaints are prominent, neuroradiological assessment should evaluate both posterior cortical involvement (ventral vs. dorsal) and long-range association pathways (ILF/IFOF for ventral-biased phenotypes; SLF/parieto-frontal connections for dorsal control/manipulation).

## 5. Lesion Studies and Clinical Neuroradiology

Lesion-based evidence is uniquely valuable for neuroradiological accounts of visual mental imagery because it can inform necessity (critical nodes and connections) rather than involvement alone. In routine neurological examinations, visual mental imagery is rarely assessed systematically and is more often recognized via patient report or formal neuropsychological testing when specifically queried.

### 5.1. Clinically Actionable Neuroradiology: Reporting Checklist for Suspected Imagery Disturbance

When a patient reports imagery disturbance (e.g., loss of the “mind’s eye”, object/face imagery loss, or spatial visualization difficulty), reporting should prioritize the following:

1. Posterior cortical nodes (gray matter): Ventral occipito-temporal involvement (object imagery) vs. dorsal occipito-parietal involvement (spatial imagery).
2. Long-range pathways (white matter): ILF/IFOF for ventral-biased phenotypes; SLF/parieto-frontal connections for dorsal control/manipulation.
3. Disconnection framing: For neuroradiological reporting, tract findings are best phrased as “tract involvement consistent with disconnection” rather than as tract-specific causality.
4. Context sequences: Correlate symptoms with conventional MRI patterns (DWI/FLAIR/atrophy), and when available, consider diffusion/tractography as supportive (model-dependent) evidence.
5. Report laterality (L/R/bilateral) of ventral OT and dorsal parietal involvement; consider dominance + disconnection.

#### Hemispheric Considerations (Lateralization)

Laterality can shape imagery phenotypes and should be reported explicitly. Current evidence suggests that left ventral occipito-temporal involvement (including fusiform cortex) [53] may be particularly relevant for acquired imagery loss in some patients (e.g., lesion–network convergence), whereas parietal contributions to spatial imagery are frequently bilateral, limiting strict hemispheric inference from single-node models [17,19]. In practice, laterality should be interpreted alongside lesion extent, dominance (handedness/language), and tract-level disconnection, as unilateral lesions can produce symptoms via network disruption.

### 5.2. Etiologies and Imaging Phenotypes

#### 5.2.1. Stroke

Posterior circulation strokes (often PCA territory) are the most frequently reported cause of acquired imagery loss, with case-based reports linking occipital/occipito-temporal and occipito-parietal damage with marked reductions in voluntary imagery, including bilateral posterior cases with profound functional impact [41,42,46]. Available reports suggest that imagery loss can be persistent after posterior strokes in some cases, although systematic longitudinal evidence on recovery trajectories remains limited [41,42,46].

Acquired aphantasia has been reported across diverse neurological events, further supporting targeted posterior node/tract evaluation when imagery loss is reported [54,55].

From a reporting perspective, posterior circulation strokes with imagery complaints warrant explicit description of occipito-temporal vs. occipito-parietal involvement and consideration of disconnection when symptom severity exceeds the lesion core (see Section 5.1).

#### 5.2.2. Tumors (and Post-Treatment States)

Brain tumors affecting posterior cortices and associated pathways can produce imagery complaints via cortical infiltration, edema/mass effect, treatment-related injury (surgery/radiation), and tract disruption (particularly optic radiations and long-range association pathways; Section 4). Direct tumor-specific clinico-radiological studies focusing on imagery outcomes are limited, so tract-based interpretations here are largely mechanistic (disconnection/edema/post-treatment effects) rather than derived from systematic imagery testing in tumor cohorts. Posterior lesions and post-treatment network changes may also be associated with internally generated visual phenomena (e.g., hallucinations), which are not equivalent to voluntary imagery but highlight vulnerability of posterior representational systems [56]. In practice, pathway-aware imaging is useful: evaluation should extend beyond the resection cavity to peri-lesional edema, radiation effects, and tract integrity (optic radiations and association tracts), as these may contribute to symptoms disproportionate to focal cortical damage [57].

In practice, radiological interpretation should therefore emphasize lesion topography, peri-lesional edema/radiation change, and displacement or suspected involvement of long-range pathways, framed as disconnection-consistent when appropriate (see Section 5.1).

#### 5.2.3. Neurodegenerative Diseases (Posterior Cortical Syndromes)

Posterior cortical atrophy (PCA) offers a natural model of progressive posterior network dysfunction, with phenotypes that may be dorsal-leaning (visuospatial) or ventral-leaning (object/word/face) depending on stage and distribution [43,44,45]. In this context, imagery complaints may serve as clinically meaningful markers of posterior network compromise, particularly when routine screening underestimates higher-order deficits.

In suspected PCA or other posterior syndromes, stream-biased atrophy patterns can be reported as ventral-leaning vs. dorsal-leaning involvement to contextualize object vs. spatial imagery complaints and guide targeted cognitive assessment (see Section 5.1).

### 5.3. Lesion–Symptom Mapping and Clinico-Radiological Correlations

Beyond single-case inference, quantitative lesion–symptom mapping (e.g., VLSM) and morphometry help link damage/atrophy with imagery outcomes while accounting for lesion distribution and covariates [40,58,59,60]. Lesion–network mapping further suggests that anatomically diverse lesions associated with acquired imagery loss may converge on common functional networks (e.g., a fusiform-linked “imagery node”), supporting a network-level interpretation [53].

For interpretable clinico-radiological correlation, imagery phenotyping should combine: object-feature and spatial-transformation tasks; stage-aware designs; and subjective vividness with objective constraints (Section 3.1).

### 5.4. Practical Synthesis: Regions, Tracts, and Expected Phenotypes

**Figure 3:**
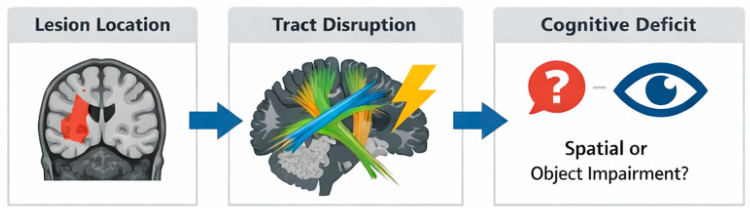
Clinico-radiological mapping linking lesion location, tract disruption, and cognitive deficit. Created by the authors for this review. Colors in the tract illustration are schematic and used only to distinguish major association pathways (e.g., ventral occipito-temporal and dorsal parieto-frontal routes); they do not represent a tract-specific atlas or a standardized color code.

To support clinico-radiological reasoning from symptoms to posterior nodes and white-matter pathways, Table 2 summarizes regions/tracts, expected imagery phenotypes (object vs. spatial), common etiologies, and dominant evidence types. This table is intended as a practical scaffold rather than a one-to-one mapping, as strategy and bilateral involvement can modulate phenotype expression. Figure 3 provides a schematic overview of the same clinico-radiological logic, linking posterior nodes and association pathways with expected imagery phenotypes across common etiologies (Figure 3).

## 6. Aphantasia and Hyperphantasia: Emerging Neuroimaging Evidence

### 6.1. Phenotyping: Object vs. Spatial Aphantasia/Hyperphantasia

Aphantasia and hyperphantasia represent extremes of voluntary imagery vividness [15,61]. Recent work proposes that vividness may fractionate into object (appearance/detail) and spatial (relations/transformations) components [3,62], although this subtyping remains provisional and not consistently validated across standardized cohorts [61,62,63,64,65]. Behavioral findings (e.g., reduced object detail with preserved spatial layout in drawing-from-memory task) support an object-selective profile in at least a subgroup [63]. For neuroradiological relevance, phenotyping should include at least one object-feature task and one spatial-transformation task alongside subjective ratings to avoid conflating components [61,62,63,64].

### 6.2. fMRI and rs-fMRI Findings

Current evidence supports a network-level account: vividness relates to how effectively higher-order systems engage posterior visual representations, but findings are largely correlational [37,38,39]. In acquired imagery loss, lesion–network mapping suggests that diverse lesions may converge on a common network linked to a fusiform “imagery node” [53]. Early visual cortex (V1/EVC) involvement appears conditional, most evident when tasks require fine-grained detail, which may explain mixed results across paradigms [30,65].

### 6.3. Clinically Actionable Questions

For clinical interpretation and neuroradiological reporting, the most actionable questions are:

(1) Whether deficits are predominantly object- vs. spatial-biased;
(2) Whether imaging suggests network/disconnection mechanisms (including long-range pathways supporting top-down access);
(3) Whether altered vividness reflects trait-like connectivity (rs-fMRI) vs. task-dependent recruitment (task fMRI) [37,38,39,53,61,63,64,65].

## 7. Clinical and Neuroradiological Implications

Visual mental imagery disturbances can be an early and functionally relevant marker of posterior network dysfunction that may be missed by routine perceptual screening. Patients may report loss of the “mind’s eye,” reduced ability to visualize faces/objects, or difficulty imagining spatial layouts and transformations; such complaints occur after posterior strokes [41,42,46], in posterior cortical syndromes (e.g., PCA) [43,44,45], and in acquired aphantasia phenotypes across neurological disorders [55]. Recognizing these symptoms can guide targeted neuropsychological testing (object vs. spatial imagery) and refine ventral–dorsal localization hypotheses [3,4,5,6,7,8,62,63].

### 7.1. Why It Matters in Practice

Imagery complaints often map onto stream- and network-level vulnerabilities: object imagery loss is more consistent with ventral occipito-temporal compromise, whereas spatial imagery impairment aligns more with dorsal occipito-parietal dysfunction and parieto-frontal control disruption [3,4,5,6,7,8,17,18,19]. A neuroradiology-forward interpretation can help explain cases where basic perception appears relatively preserved but internal visualization is profoundly impaired, consistent with disconnection or network dysfunction rather than focal cortical damage alone [47,48,53]. For example, a patient with relatively preserved basic perception but a new, specific complaint of lost object imagery (“cannot picture faces/objects”) can shift the working interpretation toward ventral occipito-temporal network involvement or tract-level disconnection, prompting more targeted structural/diffusion assessment than a purely perceptual complaint would. This framing is also relevant for counselling and rehabilitation, given impacts on everyday activities such as navigation, planning, and technical design.

### 7.2. Potential Biomarkers (Gray Matter, White Matter, and Connectivity)

Clinically useful biomarkers are likely multi-level (nodes + edges):

1. Gray matter: Posterior lesions/atrophy in occipital, occipito-temporal, and occipito-parietal regions; stream-biased atrophy patterns in posterior syndromes [43,44,45], with lesion–symptom mapping (e.g., VLSM) supporting structure–deficit links [40].
2. White matter: Tract integrity supporting top-down access and stream communication (e.g., ILF/IFOF for ventral-biased phenotypes; SLF for dorsal manipulation/control) [12,13,49,50], consistent with disconnection-based symptom expression [47,48].
3. Connectivity: Network coupling (fronto-parietal ↔ visual) associated with vividness extremes and acquired imagery loss, with task fMRI probing state-dependent recruitment and rs-fMRI indexing trait-like organization [37,38,39,53].

Overall, combined GM + WM + connectivity profiles are likely more informative than single markers.

### 7.3. Recommendations for Future Protocols and Multimodal Approaches

To improve clinical sensitivity and mechanistic specificity, future neuroradiological and clinical research protocols should prioritize the following:

Stream-aware phenotyping: Include at least one validated object-feature imagery task and one spatial transformation imagery task (to distinguish object vs. spatial imagery impairment and avoid over-reliance on appearance-weighted questionnaires) [3,62,63].

Stage-aware task fMRI: Separate imagery generation, maintenance, and transformation within event-related or mixed designs to map deficits to access/control vs. representational fidelity [1,17,18,19,31].

Tract-informed structural imaging: Pair high-quality structural MRI with diffusion imaging/tractography targeting ILF/IFOF (ventral access/integration) and SLF/parieto-frontal connections (dorsal manipulation/control), particularly in patients whose symptoms appear disproportionate to cortical injury [12,13,49,50].

Network-level inference: Incorporate lesion–symptom mapping and lesion–network mapping frameworks to distinguish local tissue loss from disconnection-driven dysfunction and to identify convergent hubs (e.g., fusiform-linked networks in acquired aphantasia) [40,53,58,59,60].

Trait + state integration: Combine rs-fMRI (trait organization) with task fMRI (state recruitment) and objective behavioral constraints (e.g., drawing or performance-validated imagery tasks) to disentangle stable predisposition from context-dependent imagery success [37,38,39,61,63,64,65].

Collectively, these recommendations support a neuroradiology-forward model in which imagery symptoms are treated as informative clinical signals of posterior network integrity and connectivity, signals that can be characterized with multimodal imaging and mapped onto ventral/dorsal stream frameworks to improve diagnostic interpretation and translational relevance.

## 8. Limitations and Future Directions

A neuroradiology-oriented synthesis of visual mental imagery is constrained by several methodological limitations in the current literature, which also define clear priorities for future work.

### 8.1. Heterogeneity of Paradigms and Outcome Measures

Imagery studies differ markedly in stimulus domains (e.g., faces, objects, and scenes), task stages (e.g., generation, maintenance, and transformation), and contrasts (imagery vs. perception vs. baseline), leading to variable recruitment of ventral, dorsal, and control systems [1,29,31]. Measures of imagery success also vary: vividness questionnaires (often biased toward appearance-based content) [32], trial-wise ratings, and objective performance indices can yield partially dissociable results, complicating cross-study comparisons and meta-analytic synthesis [33,37]. In addition, many neuroimaging studies rely on small samples and may be subject to publication bias, which can inflate apparent consistency and limit generalizability. Moreover, many tasks remain vulnerable to strategy confounds (verbal/semantic coding), particularly for surface properties such as texture, where dedicated imagery paradigms are comparatively sparse despite strong perceptual evidence for separable surface processing [23,24].

### 8.2. Need for Standardization and Subtyping (Object vs. Spatial)

The field increasingly recognizes that imagery vividness is not unitary and that phenotyping should distinguish object vs. spatial imagery components, consistent with classic dissociations and emerging aphantasia subtyping proposals [3,62,63,64]. However, there is no consensus battery that reliably separates object-feature imagery (e.g., color/form detail) from spatial transformation imagery (e.g., rotation and navigation) across cultures and clinical contexts. Standardization efforts should include validated tasks with objective constraints, harmonized reporting of imagery stage and strategy instructions, and agreed-upon core outcomes that enable comparability across laboratories and patient cohorts [58,59,60].

### 8.3. Need for Longitudinal Designs

Most imaging studies are cross-sectional, limiting inference about causality, compensation, and progression. Longitudinal approaches are especially needed in posterior neurodegenerative syndromes (e.g., PCA), where posterior network involvement evolves over time and may shift from stream-biased to mixed phenotypes [43,44,45]. Longitudinal imaging paired with repeated imagery phenotyping would clarify whether changes in vividness reflect progressive posterior cortical involvement, disconnection processes, or adaptive strategy shifts, and could help evaluate imagery as a candidate cognitive marker of posterior network integrity.

### 8.4. Multimodal Imaging and Lesions in a Unified Framework

A key future direction is integrating functional recruitment with structural substrates and lesion necessity. Multimodal designs that combine task fMRI across imagery stages [31], diffusion imaging/tractography of ventral (ILF/IFOF) and dorsal (SLF) pathways [12,13,49,50], and lesion mapping methods (e.g., VLSM and lesion–network mapping) [40,53,58,59,60] are best positioned to adjudicate whether imagery deficits reflect local representational damage, impaired top-down access, or broader network disconnection. Such integration is particularly important for acquired imagery loss and aphantasia-like phenotypes, where diverse lesion locations may converge on common networks (e.g., fusiform-linked hubs) [53] and where resting-state markers may capture trait-like vulnerability while task paradigms capture state-dependent recruitment [37,38,39,61,64,65].

Collectively, addressing these limitations will move the field toward a more precise and clinically actionable neuroradiological model of visual mental imagery, one that can explain dissociations between perception and imagery, resolve object vs. spatial subtypes, and translate imaging findings into robust clinico-radiological interpretation.

## 9. Conclusions

Visual mental imagery is best understood as a distributed brain function supported by representational systems within the ventral and dorsal visual streams and coordinated by top-down control networks. Functional neuroimaging indicates that object imagery preferentially engages ventral occipito-temporal circuits, whereas spatial imagery more strongly recruits dorsal occipito-parietal systems, with consistent evidence for task-dependent interaction between these pathways. Structural and lesion-based evidence further emphasizes that imagery disturbances can arise from focal posterior damage as well as from network disconnection affecting the white-matter routes that enable access to and integration of posterior representations.

Clinically, imagery complaints, ranging from selective object or spatial imagery impairment to acquired aphantasia-like phenotypes, should be treated as informative signals of posterior network integrity rather than as purely subjective phenomena. A neuroradiology-forward approach that combines careful phenotyping (object vs. spatial), stage-aware task paradigms, and multimodal imaging (structural MRI, diffusion tractography, and functional connectivity) offers the most promising route to resolving ongoing debates about stream independence vs. interdependence in imagery and to translating imagery research into clinically actionable interpretation. Key priorities for future work include standardized, stream-sensitive imagery tasks and multimodal designs integrating task fMRI, diffusion imaging/tractography, and lesion-based approaches.

## Figures and Tables

**Table 1:** Methods to meaning (what each method contributes to visual mental imagery).

Imaging Modality	Primary Measure	Strength for Ventral/Dorsal Question	Key Limitation	Best Use Case (Object/Spatial/Interaction)
Structural MRI (clinical)	Macroscopic lesions/atrophy; cortical involvement	Localizes cortical nodes (ventral vs. dorsal posterior cortex) relevant to object vs. spatial imagery	Limited sensitivity to microstructural disconnection and subtle network dysfunction	Clinical localization; stream hypothesis
Voxel-based morphometry (VBM)/cortical thickness	Gray matter (GM) volume or thickness differences/atrophy patterns	Quantifies stream-biased degeneration (occipito-temporal vs. occipito-parietal) and relates GM to imagery metrics	Correlational; confounded by disease stage and global atrophy	Neurodegeneration; mapping of individual differences
Voxel-based Lesion-symptom mapping (VLSM)	Voxelwise association between lesion map and behavior	Tests necessity of specific regions for object vs. spatial imagery deficits	Requires good lesion coverage and adequate sample size	Stroke cohorts; dissociation mapping
Diffusion tensor imagin/Diffusion MRI (DTI/dMRI)	White matter (WM) microstructure (e.g., fractional anisotropy/mean diffusivity (FA/MD)); tract-level integrity	Supports disconnection accounts and explains symptoms beyond cortical damage	Crossing fibers/modeling uncertainty; motion sensitivity	Pathway vulnerability; disconnection phenotypes
Tractography (ILF/IFOF/SLF, etc.)	Reconstruction of association pathways	Links stream functions with specific pathways; supports network interpretation	Method-dependent reconstructions; false positives/negatives	Pre-surgical planning; network mechanism
Task fMRI	BOLD activation during imagery tasks (generation/maintenance/transformation)	Separates content and stage (ventral object imagery vs. dorsal spatial imagery)	Strategy/compliance effects; lower SNR for imagery	Functional recruitment; stage-specific hypotheses
Resting-state fMRI (rs-fMRI)	Intrinsic functional connectivity; network organization	Trait-like markers of vividness and coupling (fronto-parietal ↔ visual)	Indirect inference; motion/physiology confounds	Vividness/aphantasia phenotyping; network biomarkers
Positron emission tomography—PET (fluorodeoxyglucose—FDG or receptor)	Metabolism or receptor binding	Reveals stream-biased hypometabolism in posterior syndromes; complements MRI	Lower spatial/temporal resolution; radiation exposure	Neurodegeneration characterization

**Table 2:** Clinico-radiological map (region/tract, stream assignment, typical imagery deficit, etiologies, and evidence).

Region/Tract	Stream (Ventral/Dorsal/Inter-Stream/Control)	Typical Deficit (Object vs. Spatial Imagery)	Common Etiologies (Stroke/Tumor/Degeneration)	Supporting Evidence Type (Lesion/DTI/fMRI/Lesion–Network)
Primary visual cortex/Early visual cortex (V1/EVC)	Inter-stream	Reduced fine-grained depictive detail; weaker vividness when tasks demand high resolution	Posterior stroke; hypoxic injury; posterior degeneration	Task fMRI; lesion/case
Occipito-temporal cortex (fusiform/inferior temporal)	Ventral	Object imagery impairment (faces/objects; appearance-based detail)	PCA territory stroke; tumors/edema; posterior cortical syndromes (PCA variants)	Lesion/case; task fMRI; lesion–network
Lateral occipital complex (LOC)	Ventral	Reduced structural object detail; impaired shape-based imagery comparisons	Occipito-temporal stroke; tumor involvement; posterior degeneration	Task fMRI; lesion inference
Color-related ventral regions (e.g., hV4)	Ventral	Impaired color imagery; reduced color-feature reinstatement	Occipito-temporal infarcts; posterior degeneration	Task fMRI
Posterior parietal cortex (IPS/SPL)	Dorsal	Spatial imagery impairment (e.g., mental rotation, transformations, and relations)	Parietal stroke; tumors; dorsal-predominant posterior syndromes	Lesion evidence; task fMRI/meta-analysis
Precuneus/medial parietal	Dorsal/integrative	Deficits in spatial context construction and integration	Posterior strokes; degeneration	Task fMRI; connectivity
Hippocampus/medial temporal	Inter-stream	Reduced coherent scene construction; impaired imagery-based episodic simulation	Neurodegeneration; hippocampal lesions	Task fMRI; neuropsychology
Inferior longitudinal fasciculus (ILF)	Ventral connectivity	Disconnection-type object imagery loss; poor access to ventral representations	Posterior WM injury; tumor tract disruption; post-treatment change	DTI/tractography; clinico-radiological
Inferior fronto-occipital fasciculus (IFOF)	Inter-stream connectivity	Reduced top-down access and integration; broad imagery weakening	Tumors/edema; surgical corridor effects; diffuse WM disease	DTI/tractography; neurosurgical mapping
Superior longitudinal fasciculus (SLF)	Dorsal connectivity	Impaired spatial manipulation/control; reduced transformation efficiency	Parieto-frontal WM lesions; tumors; post-operative disconnection	DTI/tractography; clinico-radiological
Splenium of corpus callosum	Inter-hemispheric	Reduced bilateral posterior integration; complex imagery complaints	Vascular lesions; demyelination; posterior disconnection	Structural MRI; DTI; lesion evidence
Fronto-parietal control network (e.g., DLPFC–parietal hubs)	Control	Impaired imagery generation/maintenance; reduced vividness with relatively preserved posterior cortex	Frontal lesions; diffuse network dysfunction; disconnection	Connectivity (task/rs); lesion–network

Abbreviations: ILF—inferior longitudinal fasciculus; IFOF—inferior fronto-occipital fasciculus; SLF—superior longitudinal fasciculus; IPS—intraparietal sulcus; SPL—superior parietal lobule. Laterality effects are heterogeneous; ventral imagery loss has often been reported with left occipito-temporal involvement, whereas dorsal/parietal contributions to spatial imagery are frequently bilateral; interpretation should consider dominance and disconnection.

## Data Availability

No new data were created or analyzed in this study. Data sharing is not applicable to this article.

## Abbreviations

The following abbreviations are used in this manuscript:

BOLD	blood-oxygen-level-dependent
DTI	diffusion tensor imaging
dMRI	diffusion MRI
EVC	early visual cortex
fMRI	functional magnetic resonance imaging
GM	gray matter
IFOF	inferior fronto-occipital fasciculus
ILF	inferior longitudinal fasciculus
IPS	intraparietal sulcus
IT	inferior temporal (cortex)
LOC	lateral occipital complex
PCA	posterior cortical atrophy
PET	positron emission tomography
PPA	parahippocampal place area
rs-fMRI	resting-state functional magnetic resonance imaging
RT	reaction time
SLF	superior longitudinal fasciculus
SPL	superior parietal lobule
V1	primary visual cortex
V4/hV4	visual area V4/human V4
VBM	voxel-based morphometry
VLSM	voxel-based lesion–symptom mapping
VVIQ	Vividness of Visual Imagery Questionnaire
WM	white matter
